# Dietary fat and bile juice, but not obesity, are responsible for the increase in small intestinal permeability induced through the suppression of tight junction protein expression in LETO and OLETF rats

**DOI:** 10.1186/1743-7075-7-19

**Published:** 2010-03-12

**Authors:** Takuya Suzuki, Hiroshi Hara

**Affiliations:** 1 Division of Applied Bioscience, Research Faculty of Agriculture, Hokkaido University, Sapporo, Hokkaido, Japan; 2 Graduate School of Biosphere Science, Hiroshima University, Higashi-Hiroshima, Hiroshima, Japan

## Abstract

**Background:**

An increase in the intestinal permeability is considered to be associated with the inflammatory tone and development in the obesity and diabetes, however, the pathogenesis of the increase in the intestinal permeability is poorly understood. The present study was performed to determine the influence of obesity itself as well as dietary fat on the increase in intestinal permeability.

**Methods:**

An obese rat strain, Otsuka Long Evans Tokushima Fatty (OLETF), and the lean counter strain, Long Evans Tokushima Otsuka (LETO), were fed standard or high fat diets for 16 weeks. Glucose tolerance, intestinal permeability, intestinal tight junction (TJ) proteins expression, plasma bile acids concentration were evaluated. In addition, the effects of rat bile juice and dietary fat, possible mediators of the increase in the intestinal permeability in the obesity, on TJ permeability were explored in human intestinal Caco-2 cells.

**Results:**

The OLETF rats showed higher glucose intolerance than did the LETO rats, which became more marked with the prolonged feeding of the high fat diet. Intestinal permeability in the OLETF rats evaluated by the urinary excretion of intestinal permeability markers (Cr-EDTA and phenolsulfonphthalein) was comparable to that in the LETO rats. Feeding the high fat diet increased intestinal permeability in both the OLETF and LETO rats, and the increases correlated with decreases in TJ proteins (claudin-1, claudin-3, occludin and junctional adhesion molecule-1) expression in the small, but not in the large intestine (cecum or colon). The plasma bile acids concentration was higher in rats fed the high fat diet. Exposure to bile juice and the fat emulsion increased TJ permeability with concomitant reductions in TJ protein expression (claudin-1, claudin-3, and junctional adhesion molecule-1) in the Caco-2 cell monolayers.

**Conclusion:**

Excessive dietary fat and/or increased levels of luminal bile juice, but not genetic obesity, are responsible for the increase in small intestinal permeability resulting from the suppression of TJ protein expression.

## Background

Obesity is associated with a cluster of metabolic disorders such as diabetes, hyperlipidemia, and cardiovascular diseases. The high prevalence of obesity and type 2 diabetes is becoming a serious socioeconomic and clinical problem in developed countries [1]. It has been reported that between 1/4 - 1/3 of the population in the United States can be classified as obese. Understanding the origin and features of obesity and diabetes is crucial for limiting prevalence. It is known that obesity and diabetes are the results of a complex interaction between genetic and environmental factors. Among the environmental factors, preference for a fat-enriched diet, excessive calorie intake, and a sedentary life style increase the occurrence and promote the progression of metabolic disorders.

Recently, obesity and diabetes have been characterized by low-grade chronic systemic inflammation [2], and an innovative hypothesis was proposed: this systemic inflammation is closely linked to the plasma endotoxemia resulting from increased intestinal permeability in obese animals [3,4]. Cani et al. [4] demonstrated that feeding a high fat diet increased intestinal permeability in mice, which was normalized by simultaneous antibiotic treatment. They suggested that the high fat-induced changes in the gut microflora increased intestinal permeability. Meanwhile, Brun et al. [5] reported that genetic obesity models, leptin-deficient (ob/ob) and leptin receptor-deficient (db/db) mice, displayed similar increases in intestinal permeability although these mice were fed a standard chow. Thus, the pathogenesis of the increase in intestinal permeability in obesity remains controversial.

A major determinant of intestinal permeability is the intercellular tight junctions (TJs), which are positioned around the apical end of the lateral cell membrane of intestinal epithelial cells [6]. TJs are organized by specific interactions between a wide spectrum of proteins, and three integral transmembrane proteins, occludin [7], claudin [8], and junctional adhesion molecule (JAM) [9], have been identified. These interact with other intracellular plaque proteins such as Zonula Occludens (ZO)-1, ZO-2, ZO-3, cingulin, and 7H6, which in turn anchor the transmembrane proteins to the actin cytoskeleton [6]. It is known that the association of TJ proteins with the perijunctional actin cytoskeleton ring is vital for the maintenance of TJ structure and function [10]. Stimuli, such as nutrients and cytokines, have been reported to influence not only the expression of TJ proteins but also their association with the actin cytoskeleton [11].

The aim of the present study is to explore the pathogenesis of the increase in intestinal permeability in obesity and diabetes. The effect of high-fat feeding on intestinal permeability and TJ protein expression/cytoskeletal association was investigated in another obesity model, OLETF (Otsuka Long-Evans Tokushima Fatty) rats, and the lean counter strain, LETO (Long-Evans Tokushima Otsuka) rats. In addition, based on the results from the animal study, the influences of fat and bile juice on the high-fat feeding-induced increase in TJ permeability were examined using human intestinal Caco-2 cells.

## Methods

### Chemicals

Rabbit anti-claudin-1, claudin-3, and JAM-1 and horseradish peroxidase (HRP)-conjugated mouse anti-occludin were purchased from Zymed Laboratories (San Francisco, CA). Mouse anti-β-actin and HRP-conjugated anti-mouse and -rabbit IgG were purchased from Sigma (St. Louis, MO). Cell culture reagents and supplies were purchased from Invitrogen (Carlsbad, CA). All other chemicals were obtained from Wako Pure Chemical Industries (Osaka, Japan).

### Animals and diets

Male Otsuka Long-Evans Tokushima Fatty (OLETF; n = 18) rats and the lean counter strain, Long-Evans Tokushima Otsuka (LETO; n = 18) rats, were kindly provided by Otsuka Pharmaceutical Co., Ltd. (Tokushima, Japan; 4 weeks old). Rats were housed in individual cages in a room with controlled temperature (23 ± 1°C), relative humidity (55 ± 5 %), and lighting (lights on from 8:00 to 20:00) throughout the study. Body weight and food intake were measured every 2nd day. The rats had free access to tap water and a standard diet (AIN-93G formula, Table 1) for an acclimation period of 1 week. The OLETF and LETO rats were each divided into 2 groups, the standard and high fat diet groups, on the basis of body weight (Table 1). The high fat diet contained 7% soybean oil and 23 % lard by weight. Rats had free access to each experimental diet for 16 weeks. At the end of the experiment, blood was drawn from the abdominal aorta under anesthesia (Nembutal: sodium pentobarbital, 40 mg/kg body weight; Abbott Japan Co., Ltd., Tokyo, Japan) for the measurement of tumor necrosis factor (TNF)-α, interferon (IFN)-γ, leptin, and total bile acids, and rats were then killed by exsanguination. The mucosa of the small intestine, cecum, and colon were scraped using a glass slide and subjected to preparations of whole cell extract and the detergent-insoluble fraction as described below. The liver and fat pads (mesenteric, epididymal and retroperitoneal) were removed and weighed. The mesenteric fat pad consisted of adipose tissue surrounding the gastrointestinal tract from the gastro-oesophageal sphincter to the end of the rectum. The retroperitoneal fat pad was taken as the distinct deposit on the back of abdominal wall including that around each kidney. This study was approved by the Hokkaido University Animal Committee, and the rats were maintained in accordance with the Hokkaido University guidelines for the care and use of laboratory animals (Approval number: 08-0137).

**Table 1:** Composition of test diets

Ingredient	Standard diet	High fat diet
	
	g/kg diet	
Casein^1^	200	200
Corn starch^2^	429.5	199.5
Dextrin^3^	100	100
Sucrose^4^	100	100
Soy bean oil	70	70
Lard	0	230
Choline bitartrate	2.5	2.5
L-cystine	3	3
Mineral mixture^5^	35	35
Vitamin mixture^5^	10	10
Cellulose^6^	50	50

^1 ^Casein (ALACID; New Zealand Daily Board, Wellington, New Zealand).

^2 ^Corn Starch (α-Corn Starch; Chuou Shokuryo Co., Ltd., Inazawa, Japan).

^3 ^Dextrin (TK-16; Matsutani Chemical Industry Co., Ltd. Itami, Japan).

^4 ^Sucrose (Nippon Beet Sugar Manufacturing Co., Ltd., Tokyo, Japan).

^5 ^Mineral and vitamin mixtures were prepared according to the AIN-93G formulation.

^6 ^Crystallized cellulose (Ceolus PH102; Asahi Chemical Industry, Tokyo, Japan).

### Oral glucose tolerance test (OGTT)

Oral glucose tolerance tests were performed 2, 8 and 15 weeks after the onset of feeding as described previously [12]. Briefly, the rats were fasted for 8 h before the administration of an oral glucose load (2 g/kg body weight; 200 g/L solution). Blood samples were collected from the tail vein at 0 (before administration), 15, 30, 60, and 120 min after administration of the glucose load. Blood samples were immediately centrifuged (1300 × g for 15 min at 4°C) and the plasma was separated. Plasma glucose concentration was assayed by an enzymatic method using a commercially available kit (Glucose CII Test Wako, Wako Pure Chemical Industries). The area under the glucose curve (AUC) was then calculated. Plasma insulin concentration on 15 weeks was assayed by ELISA (Rat Insulin ELISA KIT, Shibayagi, Gunma, Japan). Homeostasis model assessment insulin resistance (HOMA-IR) was calculated as an indicator of insulin resistance on 15 weeks according to the formula: HOMA-IR = Fasting glucose (mM) × Fasting insulin (μU/mL)/22.5. Plasma triglyceride concentration at 0 min was also measured using a commercially available kit (TG-EN Kainos, Kainos Laboratories, Tokyo, Japan).

### Plasma total bile acid, TNF-α, IFN-γ, and leptin concentrations

The plasma was separated from the blood collected from the abdominal aorta at the end of the experiment. The total bile acid concentration in the plasma was assayed by an enzymatic method using a commercially available kit (TBA Test Wako, Wako Pure Chemical Industries). TNF-α, IFN-γ, and leptin concentrations were assayed by ELISA (Rat TNF-α US ELISA kit, Invitrogen; Rat IFNγ ELISA kit, Pierce Biotechnology, Rockford, IL; Rat leptin ELISA kit, LINCO Research, St. Charles, MO).

### Intestinal permeability test

Intestinal permeability was evaluated using phenolsulfonphthalein (PSP) [13] and Cr-EDTA [14]. These markers pass through the paracellular routes, but not transcellular routes, of the intestinal epithelial cells into the blood stream and are excreted in the urine without being metabolized in the body. Phenolsulfonphthalein [5 mg/kg body weight, 1% (w/v) PSP solution] at 3 weeks and Cr-EDTA (450 μmol Cr-EDTA/kg body weight, 90 mmol Cr-EDTA/L solution) at 9 and 15 weeks were intragastrically administrated to the rats. Cr-EDTA was prepared as described previously [15]. The total urine was collected for 48 h after administration of the PSP and Cr-EDTA loads and diluted to 100 mL with deionized water. The PSP concentration was colorimetrically determined at a wavelength of 560 nm after the addition of sodium hydroxide (final concentration, 0.4 M) to the diluted urine. For the measurement of Cr-EDTA, ammonium chloride (final concentration, 0.2 g/L) and perchloric acid (final concentration, 3.5%) were added to the diluted urine and Cr concentrations in the supernatant obtained upon centrifugation (5,000 × g for 15 min at 4°C) were measured by atomic absorption spectrophotometry (AA-6400F; Shimadzu Corporation, Kyoto, Japan). The urinary PSP and Cr-EDTA excretions ratios were expressed as a percentage of the amounts administered.

### Immunofluorescence

Segments of the small intestine, cecum, and colon were embedded in OCT compound (Sakura Finetek Japan Co. Ltd., Tokyo, Japan) after fixation with 4 % paraformaldehyde in PBS. Frozen sections (7 μm in thickness) were prepared on glass slides and washed with PBS. The sections were blocked in 10 % normal goat serum and incubated for 16 h at 4°C with rabbit polyclonal anti-claudin-3, followed by incubation for 1 h with goat AlexaFluor 488-conjugated anti-rabbit IgG and rhodamine-conjugated phalloidine. The sections were preserved in a mounting medium containing DAPI (ProLong^®^Gold Antifade Reagent, Invitrogen), and the fluorescence was visualized using a Leica FW4000 fluorescence microscope (Leica Microsystems, Germany).

### Experiments in intestinal epithelial Caco-2 cells

To explore the effects of rat bile juice and fat on intestinal TJ integrity, human intestinal Caco-2 cells were used (HTB-37; American Type Culture Collection, Rockville, MD). The Caco-2 cells were propagated and maintained under standard cell culture conditions as described previously [11]. The cells were seeded into permeable polyester membrane filter supports (Transwell, 12 mm diameter, 0.4 μm pore size; Corning Costar Co., Cambridge, MA) at a density of 0.25 × 10^6 ^cells/cm^2^. Cultures were used between passage 40 and 50, and the medium was refreshed every 3 days.

Rat bile juice was collected from 3 male Wistar/ST rats (7 weeks old; SLC Japan, Shizuoka, Japan) fed the high fat diet for at least one week. Briefly, a silicon cannula (Silascon SH No.00; Kaneka Medix, Osaka, Japan) was implanted in the rat bile duct and the bile juice was spontaneously collected for 3 h under anesthesia (sodium pentobarbital, 40 mg/kg body weight). The total bile acid concentration in the rat bile juice collected was quantified using the commercially available kit described above. An intravenous fat emulsion (10% Intralipid^®^; TERUMO, Tokyo, Japan) was used as the fat source and contained 10 % (w/v) soybean oil, 1.2 % (w/v) lecithin, and 2.25 % (w/v) glycerol.

Intestinal TJ integrity was evaluated by measurement of transepithelial electrical resistance (TER) and unidirectional flux of lucifer yellow (LY) in Caco-2 cell monolayers in Transwell filter supports [11]. The rat bile juice [0, 1.0 2.5, 5.0 10, and 20 % (v/v)] and fat emulsion [0, 0.1, 0.25, 0.5, and 1 % fat (w/v)] were administrated to the apical wells on day 12 and the cells were maintained for 72 h. The effect of the combination of the bile juice (10 %) and fat emulsion (0.5%) on the TJ permeability was also examined. The lecithin and glycerol concentrations were adjusted to 0.12 % and 0.225% in the apical solutions of all treatments in the series of experiments using the fat emulsion. The medium was refreshed every day. TER was measured every 24 h after administration of the test agents using a Millicell-ERS system (Millipore, Bedford, MA). Lucifer yellow (LY; 100 μmol/L), a paracellular marker, was injected into the apical wells at 69 h post administration of the bile juice and fat emulsion, and the flux into the basal wells was assessed for 3 h. The concentration of LY in the basal solution was determined by fluorescence measurement (FP-550; JASCO International Co., Ltd., Tokyo, Japan). Whole extract of cells incubated with the bile juice (0, 10, and 20 %) and fat emulsion (0, 0.5, and 1 %) for 72 h was prepared for immunoblot analysis of TJ proteins as described below.

### Preparation of whole cell extract and the detergent-insoluble fraction

Whole cell extract and the detergent-insoluble fraction of the rat intestinal mucosa and Caco-2 cells were prepared as described previously [11,16], with the latter corresponding to the TJ proteins associated with the actin cytoskeleton. For preparation of the whole cell extract, 50 mg of rat mucosa (small intestine, cecum, and colon) was lysed with 500 μL of RIPA buffer (1% Nonidet P-40, 0.5% sodium deoxycholate, 0.1% SDS, 150 mmol/L NaCl, 1 mmol/L EGTA, and 1 mmol/L EDTA in 25 mmol/L Tris containing protease inhibitors and phosphatase inhibitors, pH 7.5). Two hundred μL of RIPA buffer was added to the Caco-2 cells in each well after washing the cell monolayers with ice-cold PBS. To prepare the detergent-insoluble fraction, 50 mg of rat mucosa was suspended with 500 μL of lysis buffer-CS (1% TritonX-100, 5 mmol/L EGTA in 50 mmol/L Tris containing protease and phosphatase inhibitors, pH 7.5) and incubated for 15 min at 4°C. Cell lysates were centrifuged at 15,600 × g for 5 min at 4°C to sediment the high density actin-rich fraction. The pellet corresponding to the detergent-insoluble fraction was suspended in 300 μL of RIPA buffer. Protein concentrations in the lysates were measured using the BCA method (Pierce Biotechnology, Inc., Rockford, IL). The lysates were mixed with a half volume of 3× concentrated Laemmli sample buffer [17] and heated at 100°C for 5 min.

### Immunoblot analysis

Proteins (50 μg of the rat mucosa and 20 μg of the Caco-2 cells) were separated by SDS-PAGE (12 %) and transferred to polyvinylidene difluoride membranes. Membranes were blotted for occludin, claudin-1, claudin-3, JAM-1, and β-actin using specific antibodies in combination with HRP-conjugated anti-mouse IgG or anti-rabbit IgG antibodies. The blots were developed using the ECL chemiluminescence method (GE Healthcare, Buckinghamshire, UK). Quantification was performed by densitometric analysis of specific bands on the immunoblots using Image J software.

### Statistical analysis

All values are expressed as means ± SEM. Statistical analyses were performed by 1- or 2-way ANOVA (analysis of variance) or repeated measure 1- or 2-way ANOVA followed by Duncan's multiple range test. A difference with *P *< 0.05 was considered significant. Statistical analyses were performed using the general linear models procedure of the SAS program (version 6.07; SAS Institute).

## Results

### Body weight, food intake, and tissues weights

Body weights at 0 (initial), 2, 8, and 16 (final) weeks, food intake, and energy intake were all influenced by strain, and all parameters except for the initial body weight were influenced by the diet as well (*P *< 0.05, 2-way ANOVA, Table 2). The final body weight and energy intake were higher in the OLETF than in the LETO rats and were higher in the high fat diet groups than in the standard diet groups in each strain.

**Table 2:** Initial body weight, final body weight, food intake, energy intake, liver weight, and fat pad weight

		Body weights				Food intake	Energy intake	Liver weight	**Fat pad weight**^**1**^
		
		0 week	2 weeks	8 weeks	16 weeks				
Strain	Diet	g	g	g	g	g/day	cal/day	g/rat	g/rat
LETO	Standard	91.5 ± 1.9 b	189 ± 14 b	365 ± 10 d	461 ± 13 d	18.2 ± 0.4 c	71.9 ± 1.7 d	9.49 ± 0.28 c	25.0 ± 2.4 d
	High fat	89.4 ± 1.3 b	195 ± 2 b	420 ± 6 c	551 ± 28 c	15.8 ± 0.2 d	80.5 ± 1.2 c	9.74 ± 0.34 c	47.2 ± 3.1 c
									
OLETF	Standard	114 ± 1.9 a	242 ± 3 a	500 ± 6 b	655 ± 35 b	25.1 ± 0.5 a	99.5 ± 1.7 b	17.2 ± 0.67 b	76.1 ± 2.3 b
	High fat	114 ± 1.5 a	258 ± 7 a	606 ± 16 a	769 ± 49 a	22.8 ± 0.4 b	116 ± 2.5 a	22.6 ± 0.81 a	114 ± 4.3 a
									
2-way ANOVA	Strain	< 0.01	< 0.01	< 0.01	< 0.01	< 0.01	< 0.01	< 0.01	< 0.01
	Diet	0.52	0.03	< 0.01	0.06	< 0.01	< 0.01	< 0.01	< 0.01
	S × D	0.54	0.43	0.12	< 0.01	0.93	0.04	< 0.01	0.92

Each value represents mean ± SEM, n = 9. Means without a common letter differ, P < 0.05.

^1 ^Fat pad weight: sum of mesenteric, epididymal and retroperitoneal fat pads weights

Liver and total fat pad (mesenteric, epididymal, and retroperitoneal fat pads) weights were strongly influenced by both strain and diet (*P *< 0.05, 2-way ANOVA, Table 2). These 2 parameters in the OLETF rats were much higher than in the LETO rats with the total fat pad weights in the LETO rats fed the high fat diet and in the OLETF rats fed the standard and high fat diets being 1.9-, 3.0-, and 4.6-fold higher, respectively, than that in the LETO rats fed the standard diet,.

### Oral glucose tolerance test (OGTT)

**Figure 1:**
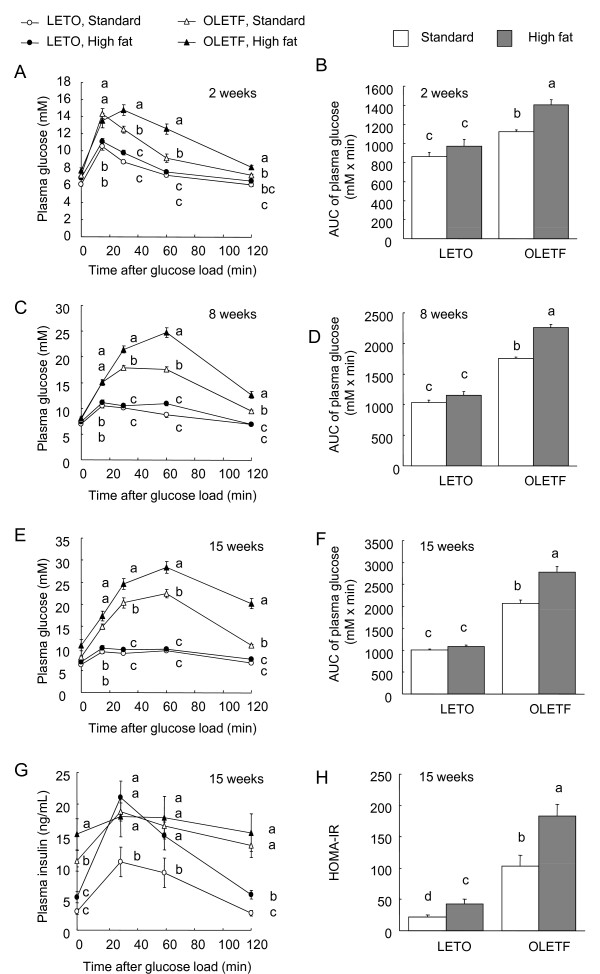
**Plasma glucose (A, C, and E) and insulin (G) concentrations, the area under the glucose curve against baseline (0 min, AUC) (B, D, and F), and HOMA-IR (H) in the OLETF and LETO rats fed the standard and high fat diets in response to an oral glucose load (2 g/kg body weight)**. Oral glucose tolerance tests were performed at 2 (A and B), 8 (C and D), and 15 weeks (E-G) after the start of feeding the experimental diets. Each value represents mean ± SEM, n = 9. Means without a common letter differ,* P < 0.05*. *P* values estimated by repeated measure 2 way ANOVA and 2-way ANOVA were (A) < 0.01 for strain, < 0.01 for diet, < 0.01 for time, 0.07 for strain x diet, < 0.01 for strain x time, < 0.01 for diet x time, and < 0.01 for strain x diet x time, (B) < 0.01 for strain, < 0.01 for diet, and 0.10 for strain x diet, (C) < 0.01 for strain, < 0.01 for diet, < 0.01 for time, < 0.01 for strain x diet, < 0.01 for strain x time, < 0.01 for diet x time, and < 0.01 for strain x diet x time, (D) < 0.01 for strain, < 0.01 for diet, and < 0.01 for strain x diet, (E) < 0.01 for strain, < 0.01 for diet, < 0.01 for time, < 0.01 for strain x diet, < 0.01 for strain x time, < 0.01 for diet x time, and < 0.01 for strain x diet x time, (F) < 0.01 for strain, < 0.01 for diet, and < 0.01 for strain x diet, (G) < 0.01 for strain, < 0.01 for diet, < 0.01 for time, 0.07 for strain x diet, < 0.01 for strain x time, < 0.01 for diet x time, and < 0.01 for strain x diet x time, and (H) < 0.01 for strain, < 0.01 for diet, and 0.10 for strain x diet.

The OLETF rats showed higher glucose intolerance than did the LETO rats, which became more marked with the prolonged feeding of the high fat diet (Fig. 1). At 2 (Fig. 1A), 8 (Fig. 1C), and 15 (Fig. 1E) weeks, the plasma glucose concentrations in the OLETF rats fed the standard and high fat diets were higher than those in the LETO rats fed the same diets at 15, 30, and 60 min post glucose challenge. Within the OLETF rats, the glucose concentrations in the high fat diet group were higher than those in the standard diet group at 30, 60, and 120 min in all tests. The area under the glucose curve (AUC) was influenced by both strain and diet at 2 (Fig. 1B), 8 (Fig. 1D) and 15 (Fig. 1F) weeks (*P *< 0.05, 2-way ANOVA), and the AUC values in the OLETF rats were higher than those in the LETO rats in all tests. In the OLETF rats, the AUC in the high fat diet group was higher than that in the standard diet group in all tests. The plasma insulin concentrations in the OLETF rats fed the standard and high fat diets for 15 weeks were higher than those in the LETO rats fed the standard diet at all time points (Fig. 1G). Within the LETO rats, the insulin concentrations in the high fat diet group were higher than those in the standard diet group at 30, 60, and 120 min. The HOMA-IR was influenced by both strain and diet on 15 weeks (Fig. 1H; P < 0.05, 2-way ANOVA), and the HOMA-IR in the OLETF rats were higher than those in the LETO rats.

### Concentrations of blood glucose, triglyceride, TNF-α, IFN-γ, leptin, and bile acid

**Figure 2:**
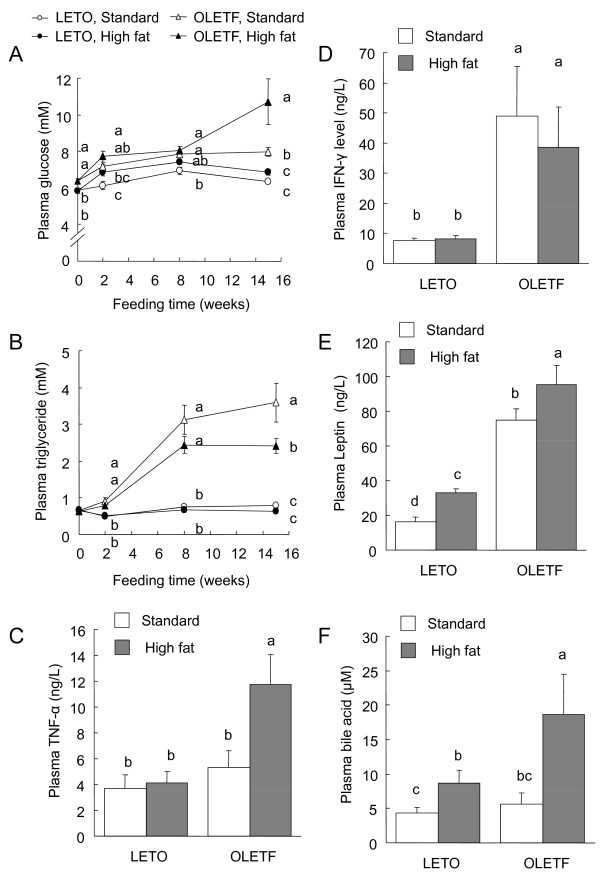
**Plasma glucose (A), triglyceride (B), TNF-α (C), INF-γ (D), leptin (E), and total bile acid (F) concentrations in the OLETF and LETO rats fed the standard and high fat diets**. Glucose and triglyceride concentrations in the plasma collected from the tail vein at 2, 8, and 15 weeks after the start of feeding the experimental diets were measured. TNF-α, INF-γ, leptin, and total bile acid concentrations in the plasma collected at the end of the experiment (at 16 weeks) were measured. Each value represents mean ± SEM, n = 9. Means without a common letter differ, *P < 0.05*. *P* values estimated by repeated measure 2 way ANOVA and 2-way ANOVA were (A) < 0.01 for strain, < 0.01 for diet, < 0.01 for time, 0.24 for strain x diet, < 0.01 for strain x time, < 0.02 for diet x time, and 0.07 for strain x diet x time, (B) < 0.01 for strain, < 0.01 for diet, < 0.01 for time, 0.02 for strain x diet, < 0.01 for strain x time, < 0.07 for diet x time, and 0.23 for strain x diet x time, (C) < 0.01 for strain, <0.03 for diet, and 0.06 for strain x diet, (D) < 0.01 for strain, <0.72 for diet, and 0.86 for strain x diet, (E) < 0.01 for strain, 0.01 for diet, and 0.82 for strain x diet, and (F) 0.10 for strain, <0.01 for diet, and 0.20 for strain x diet.

The plasma glucose and triglyceride concentrations under fasting conditions before and at 2, 8, and 15 weeks after feeding were assayed (Fig. 2A and 2B). Before the start of feeding (on 0 week), the plasma glucose concentration in the OLETF rats was higher than that in the LETO rats. At 2, 8, and 15 weeks, the glucose concentrations in the OLETF rats fed the standard and high fat diets were higher than that in the LETO rats fed the standard diet, and the glucose level in the OLETF rats fed the high fat diet was much higher than those in the other groups at 15 weeks. At 2, 8, and 15 weeks, the plasma triglyceride concentrations in the OLETF rats were much higher than those in the LETO rats. In the OLETF rats, the triglyceride concentration in the high fat diet group was lower than that in the standard diet group at 15 weeks.

The plasma TNF-α (Fig. 2C), IFN-γ (Fig. 2D), and leptin (Fig. 2E) concentrations at 15 weeks were higher in the OLETF rats than in the LETO rats (*P *< 0.05, 2-way ANOVA). The TNF-α concentration in the OLETF rats fed the high fat diet was much higher compared with those in the other groups. The IFN-γ concentrations in the OLETF rats were much higher than those in the LETO rats. The leptin concentrations were markedly higher in the OLETF rats than in the LETO rats, and feeding the high fat diet increased the leptin levels in rats of both strains (Fig. 2E). The total bile acid concentration in the plasma was increased by feeding the high fat diet in both strains, but the high fat-induced increase was much larger in the OLETF rats (Fig. 2F). There was no difference in bile acid level between the OLETF and LETO rats in the standard diet group.

### Intestinal permeability test

**Figure 3:**
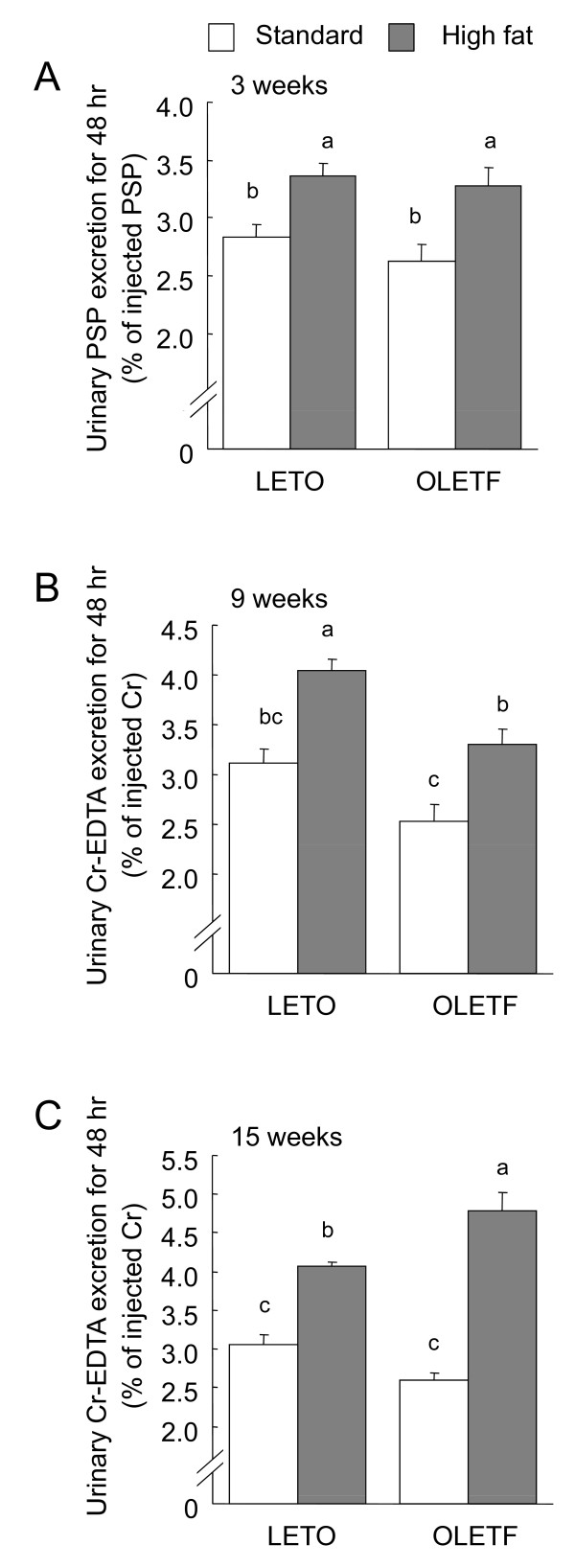
**Urinary phenolsulfonphthalein (PSP, A) and Cr-EDTA (B and C) excretion for 48 h after administration of their oral loads in the OLETF and LETO rats fed the standard and high fat diets**. The intestinal permeability tests were performed at 3 (A), 9 (B), and 15 (C) weeks after the start of feeding the experimental diets. Each value represents mean ± SEM, n = 9. Means without a common letter differ, *P *< 0.05. *P *values estimated by 2 way ANOVA were (A) 0.24 for strain, < 0.01 for diet, and 0.67 for strain × diet, (B) 0.04 for strain, < 0.01 for diet, and 0.79 for strain × diet, and (C) 0.79 for strain, <0.01 for diet, and 0.16 for strain × diet.

The urinary excretion of PSP and Cr-EDTA, intestinal permeability markers, was measured for 48 h after oral administrations (Fig. 3). The urinary PSP at 3 weeks (Fig. 3A) and Cr-EDTA at 9 (Fig. 3B) and 15 (Fig. 3C) weeks were influenced by diet (*P *< 0.05, 2-way ANOVA), and excretion levels in the high fat diet groups were higher than those in the standard diet groups in all tests. Excretion levels tended to be lower in the OLETF rats than in the LETO rats within the standard diet groups, and the OLETF rats showed lower Cr-EDTA excretion at 9 weeks as (by 2-way ANOVA). At 15 weeks, Cr-EDTA excretion in the OLETF rats fed the high fat diet was higher than that in any other group.

### TJ protein expression and cytoskeletal association in the small intestine, cecum and colon

**Figure 4:**
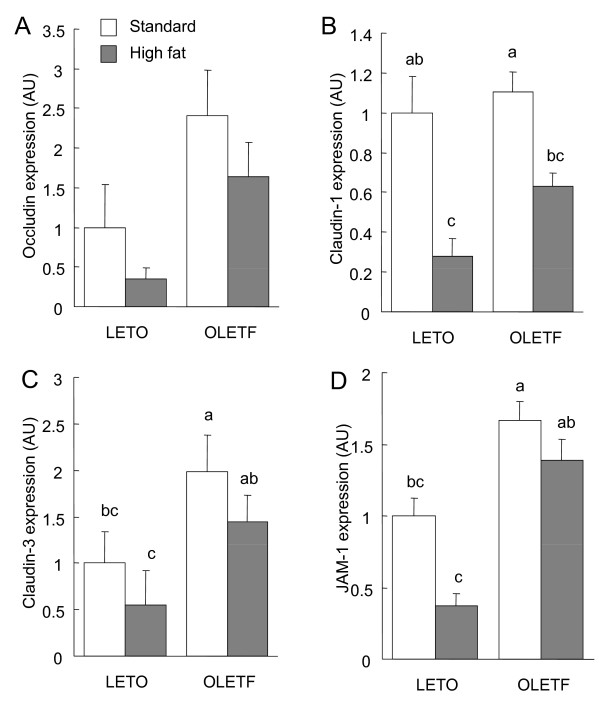
**Immunoblot analysis of tight junction (TJ) proteins in the total cell extracts of small intestinal mucosa of the OLETF and LETO rats fed the standard and high fat diets**. The mucosa was collected at the end of the experiment (at 16 weeks) and immunoblotted for occludin, claudin-1, claudin-3, junctional adhesion molecules-1 (JAM-1), and β-actin. Specific bands of occludin (A), claudin-1 (B), claudin-3 (C), and JAM-1 (D) in the whole cell extracts were quantitated by densitometric analysis. The density values were normalized to the value obtained for the LETO rats fed the standard diet. Each value represents mean ± SEM, n = 9. Means without a common letter differ, *P *< 0.05. *P *values estimated by 2 way ANOVA were (A) 0.09 for strain, 0.09 for diet, and 0.62 for strain × diet, (B) 0.06 for strain, < 0.01 for diet, and 0.31 for strain × diet, (C) 0.01 for strain, 0.04 for diet, and 0.72 for strain × diet, and (D) <0.01 for strain, <0.01 for diet, and 0.18 for strain × diet.

In the small intestine, claudin-1, claudin-3, and JAM-1 expression was influenced by diet (*P *< 0.05, 2-way ANOVA, Fig. 4), with high-fat feeding resulting in a decrease in expression. Claudin-3 and JAM-1 expression levels were also influenced by strain, and were higher in the OLETF rats than in the LETO rats (*P *< 0.05, 2-way ANOVA).

**Figure 5:**
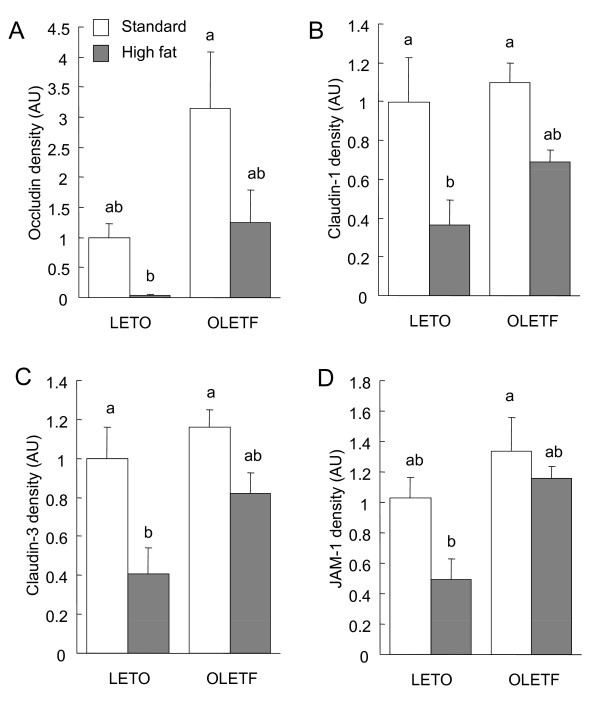
**Immunoblot analysis of tight junction (TJ) proteins in the detergent-insoluble fractions of small intestinal mucosa of the OLETF and LETO rats fed the standard and high fat diets**. The mucosa was collected at the end of the experiment (at 16 weeks) and immunoblotted for occludin, claudin-1, claudin-3, junctional adhesion molecules-1 (JAM-1), and β-actin. Specific bands of occludin (A), claudin-1 (B), claudin-3 (C), and JAM-1 (D) in the detergent insoluble fractions were quantitated by densitometric analysis. The density values were normalized to the value obtained for the LETO rats fed the standard diet. Each value represents mean ± SEM, n = 9. Means without a common letter differ, *P *< 0.05. *P *values estimated by 2 way ANOVA were (A) < 0.01 for strain, 0.02 for diet, and 0.42 for strain × diet, (B) 0.16 for strain, < 0.01 for diet, and 0.45 for strain × diet, (C) 0.03 for strain, 0.01 for diet, and 0.33 for strain × diet, and (D) 0.01 for strain, 0.04 for diet, and 0.45 for strain × diet.

The amounts of occludin, claudin-1, claudin-3, and JAM-1 in the detergent insoluble fractions, which correspond to the amounts associated with the actin cytoskeleton, in the small intestinal mucosa were influenced by diet in a manner very similar to that of their total expression (*P *< 0.05, 2-way ANOVA, Fig. 5). The amounts of TJ proteins associated with actin cytoskeleton were decreased by feeding the high fat diets in both the OLETF and LETO rats, and were all higher in the OLETF rats than in the LETO rats, except for claudin-1 (*P *< 0.05, 2-way ANOVA).

**Figure 6:**
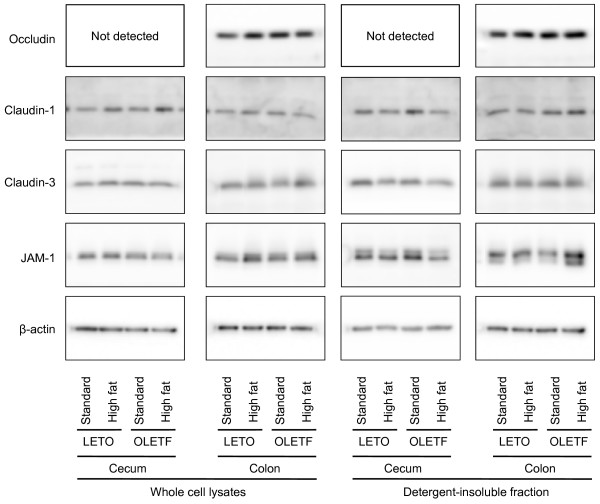
**Immunoblot analysis of tight junction (TJ) proteins in the whole cell extracts and detergent-insoluble fractions of cecal and colonic mucosa of the OLETF and LETO rats fed the standard and high fat diets**. The mucosa was collected at the end of the experiment (at 16 weeks) and immunoblotted for occludin, claudin-1, claudin-3, junctional adhesion molecules-1 (JAM-1), and β-actin. The immunoblot was representative of 8 rats.

There were no significant inter-group differences in the TJ protein expression levels or in the amounts present in the detergent insoluble fractions in the cecal and colonic mucosa (Fig. 6). No occludin was detected in the cecum when protein levels were assayed.

**Figure 7:**
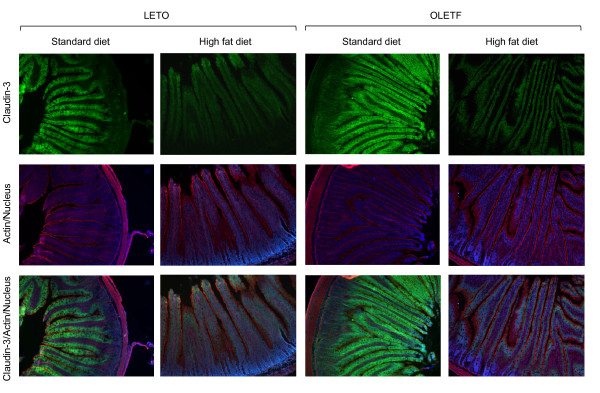
**Immunofluorescent images of claudin-3 (green), actin (red), and nucleus (blue) in the small intestine of the OLETF and LETO rats fed the standard and high fat diets**. The frozen sections (7 μm in thickness) were prepared at the end of the experiment (at 16 weeks).

Immunofluorescence microscopy showed that claudin-3 was strongly expressed in the surface epithelial cells of the small intestine, especially in the villi, but not in the crypts (Fig. 7). A higher fluorescence intensity was observed for claudin-3 in the standard diet groups than in the high fat diet groups in each strain. The fluorescence intensity in the OLETF rats fed the standard diet was higher than that in the LETO rats fed the same diet. There were no significant differences in the pattern of the actin cytoskeleton stained by rhodamine-conjugated phalloidine among the groups. In the cecum and colon, claudin-3 was expressed more in the villi than in the crypts in the surface epithelial cells however, no significant differences in fluorescence intensity were observed among the groups (data not shown).

### TJ integrity in Caco-2 cells exposed to rat bile juice and fat emulsion

The total bile acid concentration in the rat bile juice collected was 15.0 mM. Consequently, the media with 1, 2.5, 5.0, 10, and 20 % bile juice contained 0.188, 0.375, 0.75, 1.5, and 3.0 mM total bile acids, respectively.

**Figure 8:**
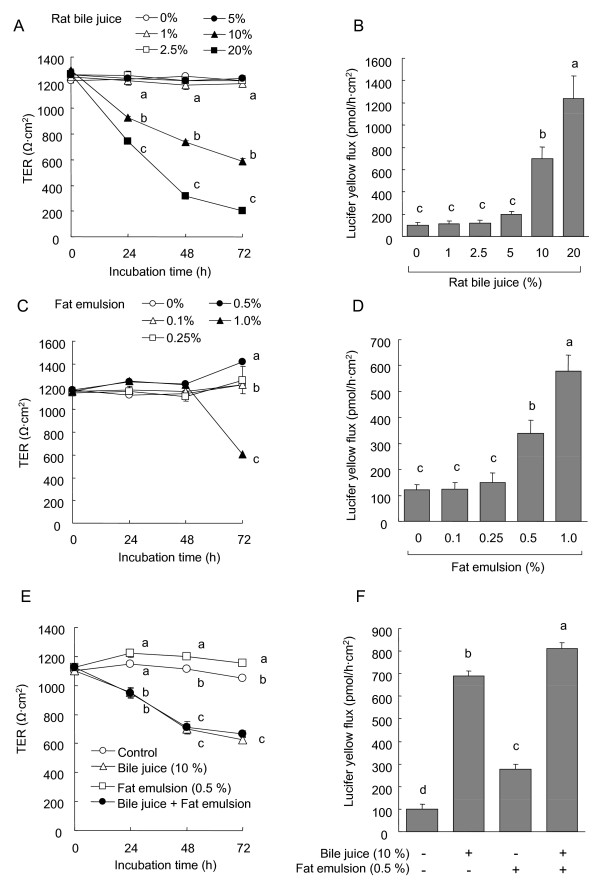
**Transepithelial electrical resistance (TER) and lucifer yellow (LY) flux across the Caco-2 cell monolayers incubated with or without rat bile juice (2.5-20 %, A and B) and fat emulsion (0.1-1.0%, C and D) for 72 h. TER (A and C) was measured at 0, 24, 48, and 72 h after the start of incubation**. LY flux (B and D) was measured for 3 h to 48 h. Each value represents mean ± SEM, n = 6. Means without a common letter differ, P* < 0.05*. *P* values estimated by repeated measure 1 way ANOVA or 1 way ANOVA were (A) < 0.01 for time, < 0.01 for treatment, and < 0.01 for time × treatment, (B) < 0.01, (C) 0< 0.01 for time, < 0.01 for treatment, and < 0.01 for time × treatment, and (D) < 0.01.

The exposure of the Caco-2 cell monolayers to 10 and 20 %, but not less than 10 %, rat bile juice periodically decreased the TER and increased lucifer yellow (LY) flux across the monolayers in a dose-dependent manner with significant differences compared with the control cells (Fig. 8A and 8B). The TER values for 10 and 20 % bile juice at 72 h were approximately 50 and 17 % of the initial values, respectively. The TER in the control cells was maintained at between 95 and 105 % of the initial value. The LY fluxes for 10 and 20% bile juice were higher than that in the control cells and were approximately 7- and 10-fold higher than the control value, respectively. There were no significant differences in TER value at any time point or in LY flux among the control cells and the cells exposed to the bile juice at less than 10 %.

The TER values in the cells incubated with or without fat emulsion (0.1, 0.25, 0.5, and 1.0 %) were sustained around the initial values until 48 h after the start of incubation, and no significant differences were found among treatments (Fig. 8C). The TER value for 1.0% fat emulsion at 72 hr was approximately 60% of the initial value. The LY fluxes for 0.5 and 1.0% fat emulsion were 3- and 5-fold higher than the control values (Fig. 8D). There were no differences in TER value at any time point or in LY flux among the control cells and the cells exposed to the fat emulsion at less than 0.5 %.

The combination of bile juice (10%) and fat emulsion (0.5%) showed the additive effects on the LY flux, but not TER (Fig. 8E and 8F). The LY flux across the cells incubated with the combination of the bile juice and fat emulsion was higher than those with each.

### Expression of TJ proteins in Caco-2 cells exposed to rat bile juice and fat emulsion

**Figure 9:**
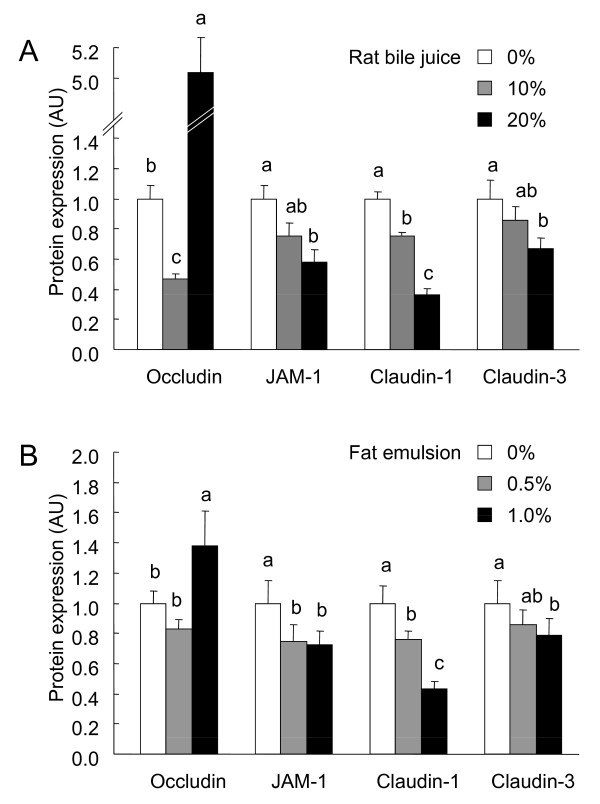
**Immunoblot analysis of tight junction (TJ) proteins in the whole cell extracts of the Caco-2 cell monolayers incubated with or without rat bile juice (10 and 20%, A and B) and fat emulsion (0.5 and 1.0%, A and C) for 72 h**. The whole cell extracts were prepared 72 h after the start of incubation and immunoblotted for occludin, claudin-1, claudin-3, junctional adhesion molecules-1 (JAM-1), and β-actin. Specific bands of occludin, claudin-1, claudin-3, and JAM-1 were quantitated by densitometric analysis. The density values were normalized to the value obtained for the control monolayers. Each value represents mean ± SEM, n = 6. Means without a common letter differ, *P *< 0.05. *P *values estimated by 1 way ANOVA were < 0.01 in each TJ protein expression in the cells treated with the rat bile juice (A) and fat emulsion (B).

Bile juice treatment decreased the expression of TJ proteins, except for occludin, in a dose-dependent manner (Fig. 9A). Occludin expression in the cells incubated with 10 and 20% bile juice were lower and higher than the control value, respectively, whereas the expression of JAM-1 and claudin-1 in cells incubated with 10 and 20% bile juice and claudin-3 in those incubated with 20% bile juice were all lower than the control value.

Occludin expression in the cells treated with 1% fat emulsion was higher than in those treated with 0 and 0.5% (Fig. 9B) whereas the expression of JAM-1, claudin-1, and claudin-3 were lower in a dose-dependent manner. The expression of JAM-1 and claudin-1 in cells treated with 0.5 and 1% fat emulsion and claudin-3 in those treated with 1% fat emulsion were all lower than the control value.

## Discussion

Recently, it was reported that mice with genetic and high fat-induced obesity and diabetes displayed increased intestinal permeability [3-5]. The endotoxemia resulting from this permeability are thought to result in the development of inflammation and metabolic disorders [3,4]. However, the relationships between the occurrence of metabolic disorders and intestinal permeability, and the mediator(s) of the increase in permeability in cases of obesity remain unclear. The present study demonstrated that obesity by itself did not increase intestinal permeability in OLETF rats, a genetic obesity and type 2 diabetes model, whereas the feeding of a high fat diet increased intestinal permeability in both OLETF and their lean counter strain, LETO, rats. The increases in intestinal permeability were associated with reductions in TJ protein expressions in the small intestine, but not the large intestines (cecum and colon). In addition, the treatment of intestinal Caco-2 cells with a fat emulsion and rat bile juice remarkably increased epithelial permeability concomitantly with reductions in the expression of some TJ proteins.

Feeding the high fat diet increased the urinary excretion of PSP and Cr-EDTA, indicators of intestinal permeability, in both LETO and OLETF rats at 3, 9, and 15 weeks after the onset of feeding. Meanwhile, the intestinal permeability in the OLETF rats fed the standard diet was lower than or comparable with that in the LETO rats fed the same diet in all permeability tests. The OLETF rats fed the standard diet presented with metabolic disorders such as obesity and glucose intolerance, and the severity of these disorders increased with age although feeding with the high fat diet exacerbated them. These results suggest that these metabolic disorders themselves do not trigger the increase in intestinal permeability in the OLETF rats, instead, the high-fat feeding is the cause of the increase in intestinal permeability. The high fat-induced increase in intestinal permeability in the OLETF rats was greater at 15 weeks than at 3 or 9 weeks, indicating that prolonged feeding of the high fat diet aggravated the increase in intestinal permeability in the OLETF rats, but not in the LETO rats. The differences found between the 2 strains suggest that factors other than dietary fat affected intestinal permeability in the OLETF rats at 15 weeks. Only the OLETF rats fed the high fat diet showed higher both TNF-α and IFN-γ levels in the plasma. It is reported that TNF-α and IFN-γ synergize to impair intestinal barrier function in the intestinal cells [18]. The simultaneous increase in the TNF-α and IFN-γ is a candidate for the greater permeability in the OLETF rats fed the high fat diet for15 weeks.

To explore the mechanisms underlying the high fat-induced increase in intestinal permeability, we evaluated the expression of TJ proteins, occludin, claudin-1, claudin-3, and JAM-1, and their association with the actin cytoskeleton in the small intestine, cecum and colon of rats. Feeding the high fat diet clearly decreased the expression of some TJ proteins and their cytoskeletal association in the small intestines of LETO and OLETF rats, although we did not find any statistical differences in occludin expression (*P *= 0.09, 2-way ANOVA) in the small intestine. The decrease in the cytoskeletal association of TJ proteins was considered to originate in the decrease in their expression due to the similarities observed. In addition, the high fat-induced reduction in claudin-3 expression in the small intestine of the LETO and OLETF rats was confirmed by immunofluorescence microscopy. These results agree with those of a previous report showing that high-fat feeding increased intestinal permeability and decreased the expression of TJ proteins in the mouse jejunum [4]. Interestingly, we did not find any high fat-induced decreases in TJ protein expression or cytoskeletal association in the cecum and colon of the LETO and OLETF rats. These results indicate that the decreases in TJ protein expression and cytoskeletal association in the small intestine are responsible for the high fat diet-induced increase in intestinal permeability in the LETO and OLETF rats. Diffusion of bacterial endotoxins into the blood stream as a result of the increase in intestinal permeability is thought to be associated with the systemic inflammation and development of metabolic disorders in obesity and diabetes [3,4,19]. Our results suggest that small intestinal bacteria contributes to the endotoxemia in the high fat-induced obesity models although we did not assess the plasma endotoxin levels and more bacteria are known to colonize the large intestines than the small intestine.

We suggest excessive dietary fat itself and increased levels of bile juice in the lumen as possible mediators of the increase in permeability in the small intestine of rats fed the high fat diet. The reasons for our suggestion are 1) the high-fat feeding increased the luminal fat concentration in both the LETO and OLETF rats, 2) the increases in plasma bile acids levels by the high-fat feeding in both the LETO and OLETF rats possibly result from their secretion into the lumen in these rats 3) the LETO rats fed the high fat diet exhibited higher intestinal permeability without any change in plasma TNF-α and IFN-γ, and 4) TJ protein expression and cytoskeletal association were not affected by the high-fat feeding in the large intestine as most of that dietary fat did not reach to the large intestine and the concentration(s) of bile juice or its components was much lower in the large intestine than in the small intestine [20]. As expected, the exposure of Caco-2 cells to the fat emulsion (0.5 and 1 %) and rat bile juice (10 and 20 %) markedly increased TJ permeability with concomitant reductions in the expression of some TJ proteins, indicating that the excessive dietary fat and bile juice negatively modulate intestinal TJ integrity. Remarkably, these results in the Caco-2 cells partially mimicked the high fat diet-induced reduction in TJ proteins in the small intestines of rats, except for occludin, whereas the bile juice at ~5% or fat emulsion at ~0.5% did not have any effects on TJ permeability in the Caco-2 cells. The effective dose of the bile juice was 10 % or more, and the medium with 10% bile juice contained 1.5 mM bile acids as the total bile acid concentration in the bile juice was 15.0 mM. The luminal concentrations of the total bile acids are reported to be ~3 mM in the small intestine, ~1 mM in the cecum, and ~0.5 mM in the colon of rats [20]. The reason why the feeding of a high fat diet did not impair TJ protein expression in the large intestines seems to be that the concentrations of bile juice and dietary fat were below the concentrations needed to be effective. The plasma bile acids were unlikely to influence the intestinal TJ permeability, because the plasma levels were much lower than luminal levels. Furthermore, the additive effect on the LY flux observed by the combination of bile juice and fat emulsion indicates that their individual effects do not interact each other although they exist together in the intestinal lumen. These results observed in the Caco-2 cells indicated that the high fat feeding-induced impairment of the TJ expressions in the small intestine of the LETO and OLETF rats was caused by the increases in the luminal fat amount and bile juice.

Some reports have examined the effects of the fatty acids and the bile acids on intestinal TJ integrity. Oleic acid, which is a major fatty acid composed of the fat emulsion used in our experiment, as well as γ-linoleic, and docosahexaenoic acids reportedly increased TJ permeability in Caco-2 cells [21,22]. In these reports, the fatty acids were suggested to penetrate the plasma membrane of the cells and disturb TJ function. Meanwhile, Raimondi et al. [23] reported that unconjugated bile acids increased TJ permeability via occludin dephosphorylation, but not via any reduction in TJ protein expression, in Caco-2 cells. The differences between the previous results and our observation may be attributed to other components in the bile juice such as bilirubin and cholesterol. In addition, bile acids are conjugated with glycine and taurine in bile juice, and these conjugations provide hydrophilicity for the bile acids, which possibly affects their biological effects. In addition, Araki et al. reported that cholic acid induced the TER decrease through the generation of reactive oxygen species in the Caco-2 cells [24]. The mechanism underlying the reduction in TJ protein expression induced by the fat emulsion and bile juice remain under investigation.

The LETO rats fed the high fat diet showed the increase in the intestinal permeability without any increases in the plasma inflammatory cytokines levels, TNF-α or IFN-γ although the increased permeability resulting in the exdotoxemia is considered to be an important factor to develop the inflammatory tone in the obesity [3]. Meanwhile, the OLETF rats fed the standard diet showed the increase in the plasma IFN-γ level without increasing the permeability. These results may indicate that the LETO rats fed the high fat diet needed longer time to show the systemic inflammation resulting from the increase in permeability and that not only the intestinal permeability but also the metabolic changes with the accumulation of body fat contribute to the development of inflammatory tone in the obesity.

As described above, the OLETF rats fed the standard diet did not exhibit any increase in intestinal permeability in comparison to the counter strain LETO rats fed the same diet, although 2 obese mouse models, the leptin-deficient (ob/ob) and leptin receptor-deficient (db/db) strains, reportedly showed increases in intestinal permeability with a concomitant reduction in the expression of TJ proteins, ZO-1 and occludin [5]. These 3 animal models (OLETF rats, ob/ob mice, and db/db mice) are resistant to the hypothalamic actions of hormones, and experience hyperphagia together with subsequent obesity and metabolic disorders. However, their molecular mechanisms are different. The OLETF rat is a spontaneous cholecystokinin-1 receptor knock out model possessing defects in the satiety action of cholecystokinin [25,26]. On the other hand, the ob/ob and db/db mice present a defect in leptin activity: the ob/ob mice express a truncated inactive form of leptin, and the db/db mice express a signaling-incompetent long isoform of the leptin receptor [27]. Some reports have demonstrated that leptin ameliorates ischemia-reperfusion-induced mucosal injuries in the small intestine of rats through the stimulation of cell proliferation and prevention of cell apoptosis [28]. Leptin may also have an important role in the expression and function of intestinal TJs similar to that of epidermal growth factor [29]. Our results showed that the OLETF rats had higher plasma leptin levels and higher TJ protein expression levels in the small intestine than did the LETO rats, both of which support this hypothesis. However, further studies are required to clarify the possible role of leptin on TJ function.

## Conclusions

The feeding of a high fat diet in rats increases intestinal TJ permeability resulting from the reduction in TJ protein expression in the small, but not in the large intestine. The possible mediators of the increase in permeability are excessive dietary fat itself and the increased levels of bile juice in the lumen. Meanwhile, the obesity and the metabolic disorders themselves were not the primary factors in the increase in intestinal permeability.

## Abbreviations

ANOVA: Analysis of variance; AUC: Area under the glucose curve; HOMA-IR: Homeostasis model assessment insulin resistance; IFN-γ: Interferon-γ; JAM: Junctional adhesion molecule; LETO: Long Evans Tokushima Otsuka; LY: lucifer yellow; OGTT: Oral glucose tolerance test; OLETF: Otsuka Long Evans Tokushima Fatty; PSP: Phenolsulfonphthalein; TER: Transepithelial electrical resistance; TJ: Tight junction; TNF-α: tumor necrosis factor-α; ZO: zonula occludens.

## Competing interests

The authors declare that they have no competing interests.

## Authors' contributions

TS and HH designed the study. TS performed all experiments, assays, and statistical analysis. TS and HH analyzed the data. TS wrote the manuscript and HH helped to write the manuscript. All authors read and approved the final manuscript.
